# Galectin 3 inhibition attenuates renal injury progression in cisplatin-induced nephrotoxicity

**DOI:** 10.1042/BSR20181803

**Published:** 2018-12-18

**Authors:** Hong-yan Li, Shen Yang, Jing-chun Li, Jian-xun Feng

**Affiliations:** 1 Division of Nephrology, Huadu District People’s Hospital of Guangzhou, Southern Medical University, Guangzhou 510800, P.R. China; 2 Division of Nephrology, Xuhui District Centeral Hospital of Shanghai, Shanghai, P.R. China

**Keywords:** acute kidney injury, apoptosis, galectin-3, modified citrus pectin, renal fibrosis

## Abstract

Nephrotoxicity is a major toxic effect in chemotherapy, which constitutes up to 60% of hospitalized acute kidney injury (AKI). Very few treatment options exist to slow the transition from AKI to subsequent chronic kidney diseases (CKD). Here, we demonstrate that galectin-3 (Gal-3), a β-galactoside binding lectin that plays an important role in kidney fibrosis and renal failure, is one of the key factors for renal injury progression. Ectopic overexpression of Gal-3 significantly decreased the viability of HEK293, simultaneously inducing of cell cycle arrest and apoptosis. However, inhibition of Gal-3, mediated by modified citrus pectin (MCP), predominantly antagonized the pro-apoptotic effects. Mice were pre-treated with normal or 1% MCP-supplemented drinking water 1 week before cisplatin injection. Analyses of serum creatinine and renal tissue damage indicated that MCP-treated mice demonstrated increased renal function and attenuated renal fibrosis after cisplatin-induced injury. MCP-treated mice also demonstrated decreased renal fibrosis and apoptosis, as revealed by masson trichrome staining and Western blot analysis of cleaved caspase-3. Additionally, the protective role of Gal-3 inhibition in the kidney injury was shown to be mediated by protein kinase C α (PKC-α), which promoted cell apoptosis and collagen I synthesis in HEK293 cells. These results demonstrated the potential Gal-3 and PKC-α as therapeutic targets for the treatment of AKI and CKD.

## Introduction

Acute kidney injury (AKI) and chronic kidney disease (CKD) are critical global health challenges [1]. The use of multiple chemotherapy drugs is a major risk factor of AKI, and is associated with high mortality and morbidity. Over the past decade, the incidence of moderate to advanced stages of CKD increased considerably [2]. However, clinical management of the diseases is a challenge and very few treatment options exist currently to slow renal fibrosis progression [3]. The transition of AKI to CDK results from uncontrolled expansion of interstitial extracellular matrix (ECM) and nephron loss [4]. Two critical mechanisms contribute to renal fibrosis following renal injury: (i) apoptosis of tubular and (ii) dysregulated modeling of tissue that stems from the imbalance between matrix degradation and matrix synthesis [4,5]. Therefore, there is an urgent need for strategies to alleviate tubular injury in parallel to reducing interstitial matrix expansion.

Galectin-3 (Gal-3) is a multifunctional protein secreted by epithelial cells, macrophages and endothelial cells. It mediates a large variety of biological processes through carbohydrate-independent mechanisms [6]. Consequently, Gal-3 has been a therapeutic target in a large array of diseases, including cancer [7], cardiovascular diseases [8], lung fibrosis [9] etc. In clinics, the role of Gal-3 as a therapeutic target in cardiovascular diseases has been demonstrated [10,11]. It is suggested that Gal-3 is a marker for fibrosis, and the level of Gal-3 is predictive of heart failure and renal dysfunctionality [12–15]. It was shown that through inhibiting Gal-3, protective effects can be achieved in renal diseases [16,17]. Recently, modified citrus pectin (MCP), a derivative of pectin affinitive to Gal-3 carbohydrate recognition domain [18], has been used as an inhibitor of Gal-3 and shown to exert ameliorating effects in renal disease [19,20]. This evidence suggests that Gal-3 inhibition is a viable strategy in the treatment of renal diseases. However, the function of Gal-3 in renal diseases remains elusive. Understanding of the role of Gal-3 in ECM remolding is also lacking. Elucidation of the mechanism of Gal-3 inhibition in kidney disease therapy is imperative to optimize clinical outcome of this treatment regimen.

Herein, the purpose of the study is to clarify the role of Gal-3 inhibitive strategy in treatment of cisplatin-induced renal injury. Our findings suggest that Gal-3 inhibition, through the regulation of protein kinase C α (PKC-α), which is a major isoform of PKC in kidneys that maintains normal renal function [21], exerts renal protective effects by alleviating kidney tissue apoptosis, and reducing collagen I synthesis.

## Materials and methods

### Animals

All animal experiments were conducted according to the guidelines of laboratory animal care and were approved by the Institutional Animal Care and Use Committee of the Huadu District People’ Hospital of Guangzhou (No.2017012). Seven-to-eight-week-old male C57BL/6 mice (*n*=5) were i.p. injected with cisplatin, dissolved in 0.9% saline at 1 mg/ml, at the dose of 20 mg/kg or saline. Mice were killed at 72 h after cisplatin injection, and kidneys were perfused and harvested. Some of the mice received the Gal-3 activity inhibitor (*n*=5), modified citrus pectin (EcoNugenics, 100 mg/kg/day) in the drinking water for the same period, while some were pre-treated with normal or 1% MCP-supplemented drinking water at 7 days prior to cisplatin injection. Each mice was given the same amount of drinking water.

### Morphological and histological evaluation

Before mice were killed, blood was collected for urea measurements, with kidneys harvested for mRNA and histological end-points analyses. Blood serum creatinine was measured by ELISA using commercial kits according to the manufacturer’s instruction (Nanjing Jiancheng). The acute tubular morphology and patterns of regeneration in the outer medulla were evaluate blindly using morphometric assessment with a computer-assisted image system, KS300 (Zeiss, Jena, Germany), and Olympus BX40F-3 microscope (Olympus Optical Company, Tokyo, Japan). The results were presented as a ratio of the injured area versus the total area. Twenty-five random fields were counted at an original magnification, covering the entire outer medulla and corresponding to an area. Patterns that indicate tubular damage included: loss of nuclear and cytoplasmatic membrane integrity, vacuolization of tubular epithelial cells, loss of brush border and presence of intratubular debris. Tubular repair or regeneration was identified by decreased epithelium layer that encloses dilated lumens, and presence of proteinaceous fluid or granular material within lumens. Ki-67, Gal3 and PKC-α expression was detected in paraffin sections by immunohistochemistry using 10 ug/ml of anti-Ki-67, Gal-3 and PKC-α antibody (Abcam), according to the manufacturer’s protocol.

### Sirius Red staining

The slides were incubated with a 0.1% Sirius Red solution dissolved in aqueous saturated picric acid for 1 h, washed in acidified water (0.5% hydrogen chloride), dehydrated and mounted with DPX Mounting. Collagen and non-collagen components were red- and orange-stained, respectively.

### Detection of cytokines and chemokines

Chemokines and cytokines in the kidney tissue were measured by enzyme-linked immunoabsorbent assay (ELISA) kits. Kits used in the present study were summarized in detail. Mouse IL-1, IL-6 (eBioscience, San Diego, CA), Mouse TNFa and MCP1 (DAKEWE, Beijing, China).

### Cells culture and treatment

HEK293, acquired from American Type Culture Collection, (ATCC, Wesel, Germany), was maintained in Dulbecco-modified essential medium with 10% fetal bovine serum, 100-U/ml penicillin and 100-U/ml streptomycin. The cells were seeded on six-well plates and grown to 80% confluence before changing to serum-free medium.

### Construction of PKC-α plasmid

Total RNA was extracted from rat kidney IM by Trizol reagent (Invitrogen, Carlsbad, CA) and reverse transcripted into cDNA. The PKC-α coding region gene was obtained by RT-PCR and cloned into mammalian expression vector pcDNA or Xenopus expression vector pGH19. The PKC-α gene and cloning orientation were verified by DNA sequencing.

### Real-time reverse transcription PCR

Total RNA from kidney tissues was isolated using the Trizol Reagent (Euromedex, Souffelweyersheim, France) and purified using the RNeasy kit (Qiagen, Hilden, Germany), according to the manufacturer’s instructions. Synthesis of cDNA was carried out using the cDNA synthesis kit in a thermocycler (Bio-Rad, California, U.S.A.). Quantitative PCR analysis was then performed using SYBR green (Bio-Rad, California, U.S.A.). Quantification of expression was achieved with MyiQ software (Bio-Rad, California, U.S.A.). Data were normalized to HPRT and β-actin levels. All PCRs were performed at least in triplicate for each experiment.

### Western blot

Protein extraction was achieved using RIPA buffer supplemented with proteinase inhibitor cocktail. Protein quantification was performed using the BCA assay (Bio-Rad). Protein lysates of equal amounts were loaded and separated on SDS-polyacrylamide gels in Tris/SDS buffer, followed by transferring onto nitrocellulose membranes. Blotting was performed according to standard procedures with primary antibodies against PKC-α, galectin-3, collagen I, cleaved-caspase3, fibronection and GAPDH (Abcam) overnight. Following this, appropriate fluorescence-conjugated secondary antibodies were added. Images of the blot were acquired using an Odyssey IR scanner, and protein band intensities were quantified using NIH Image/J software.

### Statistical analysis

The data were expressed as the means ± SD. The results were statistically analyzed using a one-way or two-way analysis of variance (ANOVA). Comparisons between two groups were analyzed using Student’s *t* test. Differences with *P*<0.05 were considered statistically significant.

## Results

### Effects of pharmacological inhibition of Gal-3 on renal function in mice after cisplatin injection

**Figure 1:**
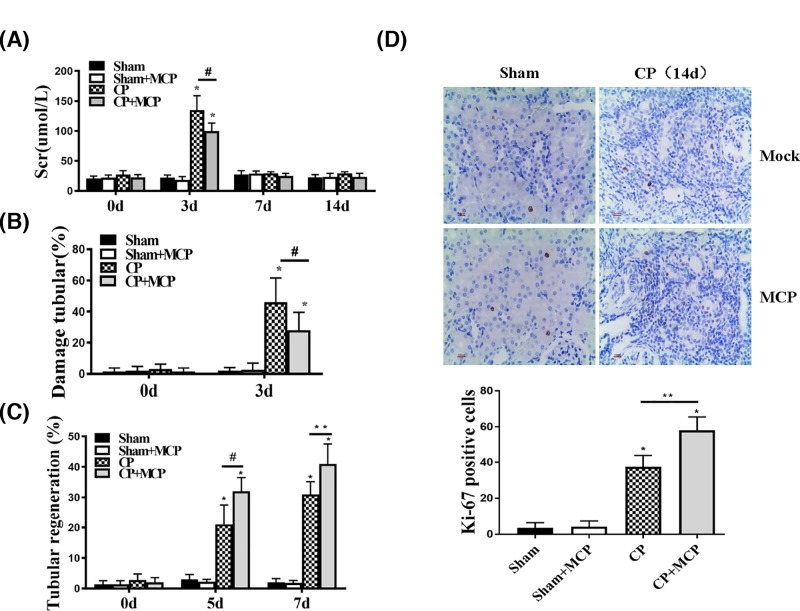
Effect of pharmacological inhibition of Galectin-3 (Gal-3) on renal function in mice after cisplatin injection (**A**) Blood serum creatinine in mice. *P<0.05 vs sham, #*P*<0.05 CP vs CP+MCP, *n*=5. (**B**) Tubular damage percentages in mice subjected to the CP injection. *P<0.05 vs sham, #*P*<0.05 CP vs CP+MCP, *n*=5. (**C**) Tubular regeneration percentages in mice subjected to the CP injection. *P<0.05 vs sham, #*P*<0.05 CP vs CP+MCP, *n*=5. (**D**) Representative sections of kidney stained for Ki-67 from different groups of mice.

To characterize the effects of Gal-3 on renal protection, renal function was evaluated by measuring blood serum creatinine (Scr) levels at 0, 3, 7 and 14 days after cisplatin (CP group) injection (20 mg/kg) or saline (sham group). Some mice in the two groups were treated with 1% MCP (CP+MCP group or sham+MCP group) before CP or saline injection. At day 3, mice that received only CP exhibited the highest Scr levels, and mice fed with 1% MCP before CP injection demonstrated significant reduction in Scr. And there was no statistical difference in Scr levels between the CP+MCP group and sham groups at day 3. At 7 and 14 days after injection, the Scr levels in all groups demonstrated similar levels (Figure 1A). Morphometric analyses confirmed renal protection in MCP+CP group animals. Three days after CP injection, animals with CP injection had the highest values of ATN, while MCP improved the effect of CP-induced tubular damage (Figure 1B). Meanwhile, animals in MCP+CP group exhibited more tubular regeneration at all-time points studied (Figure 1C). No significant ATN or tubular regeneration was found in each group of sham-treated mice. To confirm the tubular repair induced by MCP, we performed Ki-67 staining, which indicated that animals treated with MCP+CP demonstrated the highest Ki-67 levels, suggesting intensive regeneration (*P*<0.05). While regeneration was also seen in mice treated with only CP (*P*<0.05), the Ki-67 staining was relatively lower (*P*<0.01)

### Reduction of tubulointerstitial injury by Gal-3

**Figure 2:**
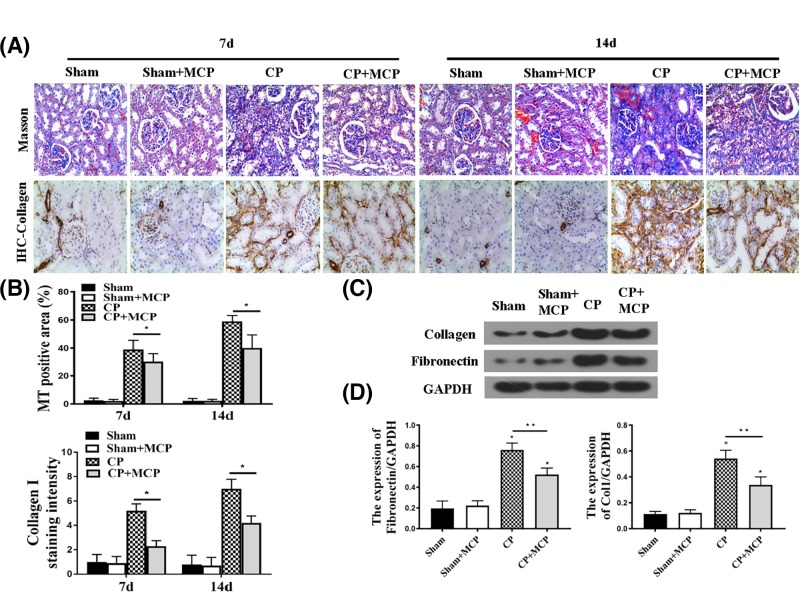
The effect of pharmacological inhibition of Gal-3 on tubulointerstitial injury (**A**) Masson trichrome (MT) and Sirius Red staining to assess the cumulative CP-induced toxicity for this model, some mice (*n*=6) were killed at 7 weeks after CP injection, and some mice (*n*=6) were killed at 14 weeks (CP: 20 mg/kg). (**B**) Representative of MT positive area (%) in each group. *P<0.05 CP vs CP+MCP; *n*=5. (**C** and **D**) Representative Western blot gel documents and summarized data shown the protein levels of collagen I and Fibronectin in the kidney. *P<0.05 vs sham; **P<0.05 CP vs CP+MCP, *n*=5.

To confirm the renal protective effects of Gal-3, pathological analysis was conducted at 7 and 14 days following CP injection to examine renal interstitial fibrosis. As expected, CP injected mice revealed moderate renal interstitial fibrosis. In contrast, MCP ameliorated renal interstitial fibrosis both at 7 and 14 days, as revealed by Sirus Red staining, masson trichrome staining and collagen staining (Figure 2A,B). Concomitantly, the protein levels of collagen I and Fibronectin in the kidney were higher in CP group at day 7 than those in mice treated with MCP (Figure 2C,D). We also analyzed IL-1β levels in different groups (Supplementary Figure S1). The kidney injury attenuation exerted by MCP was verified by that while cisplatin treatment increases IL-1β protein (Supplementary Figure S1A) and mRNA levels (Supplementary Figure S1B), suggesting increased inflammation, MCP reduced IL-1β levels. These data support the efficacy of Gal-3 inhibition on protecting again kidney injury.

### PKD3 and Gal-3 up-regulation induced by MCP promotes cell apoptosis and collagen I synthesis

**Figure 3:**
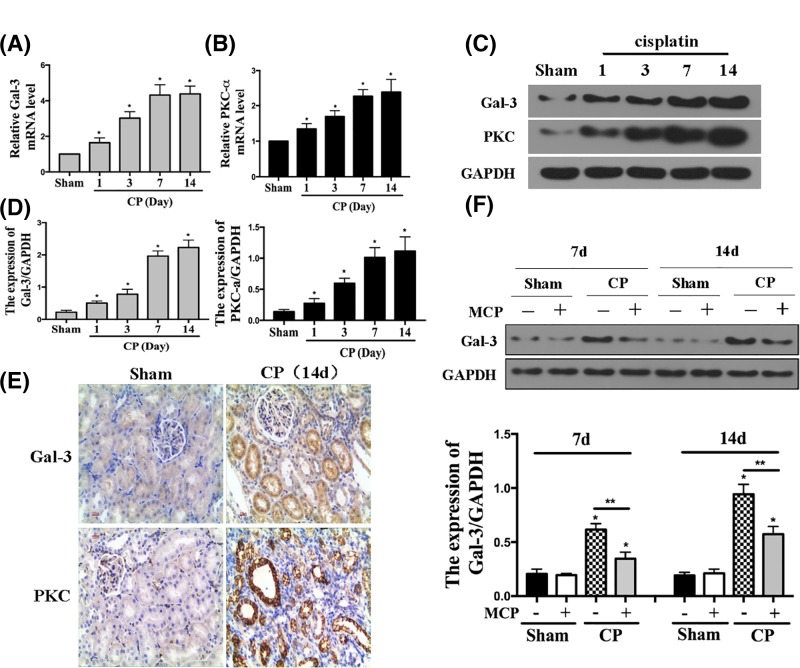
The expression of PKC-α and Gal-3 in the kidney after cisplatin treatment (**A**) Relative mRNA levels of Gal-3 in the kidney after CP injection. *P<0.05 vs sham, *n*=5. (**B**) Relative mRNA levels of PKC-α in the kidney after CP injection. *P<0.05 vs sham, *n*=5. (**C** and **D**) Representative Western blot gel documents and summarized data showing the protein levels of Gal-3 and PKC-α in the kidney. *P<0.05 vs sham, *n*=5. (**E**) Representative sections of kidney stained for Gal-3 and PKC-α from different groups of mice. (**F**) Representative Western blot gel documents and summarized data showing the protein levels of Gal-3 in the kidney.

To elucidate the mechanism of the MCP in renal protection, we evaluated the expression of PKD3 and Gal-3 using qRT-PCR (Figure 3A,B) and Western blot (Figure 3C,D) analyses, which showed that Gal-3 and PKC-α levels were time dependently up-regulated in the kidney after CP treatment followed by different time points. To confirm the change of Gal-3 and PKC-α levels following CP treatment, we performed immunohistochemical staining and Western blot analyses of Gal-3 and PKC-α, which demonstrated that MCP indeed increased Gal-3 and PKC-α expression (Figure 3E,F).

**Figure 4:**
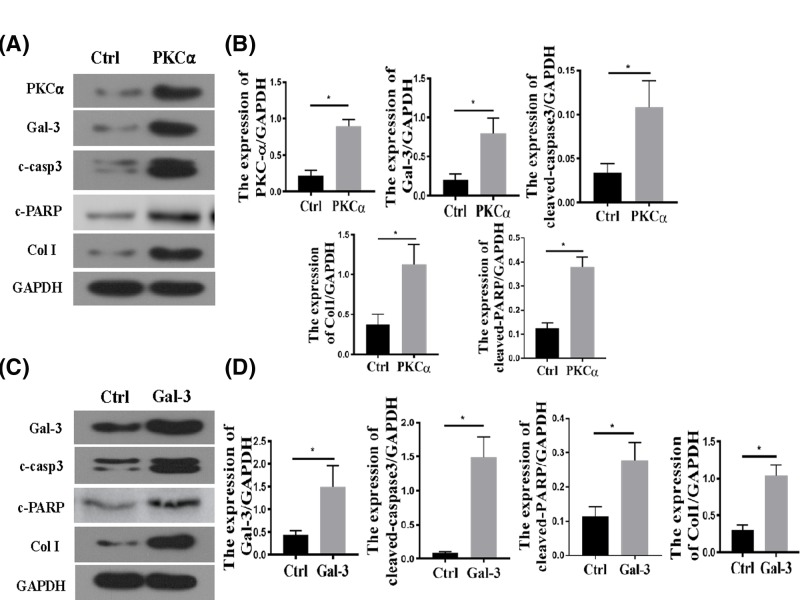
PKC-α and Gal-3 promote cell apoptosis and collagen I synthesis (**A** and **B**) The protein levels and representation of PKC-α, Gal-3, cleaved-caspase3, cleaved-PARP and collagen I in the HEK 293 cells; *P<0.05. (**C** and **D**) The protein levels and representation of Gal-3, cleaved caspase-3 (c-cap3), cleaved-PARP and collagen I in the HEK 293 cells; *P<0.05.

Consistently, we demonstrated that PKC-α overexpression induced by transferring PKC-α overexpression plasmid into HEK293 cells led to increased cell apoptosis and ECM synthesis as evidenced with increased cleaved-caspase-3 and collagen I expression levels in Western blot analysis. Interestingly, PKC-α also increased the Gal-3 levels (Figure 4A,B). As shown in Figure 4C,D, Gal-3 overexpression also increased the cleaved caspase-3 and collagen I expression. Collectively, these results suggested that PKC-α and Gal-3 overexpression are associated with increased cell apoptosis and collagen I synthesis in kidney, which qualify them as potential therapeutic targets in cisplatin-induced kidney injuries.

### PKC-α mediated cisplatin induced Gal-3 expression

**Figure 5:**
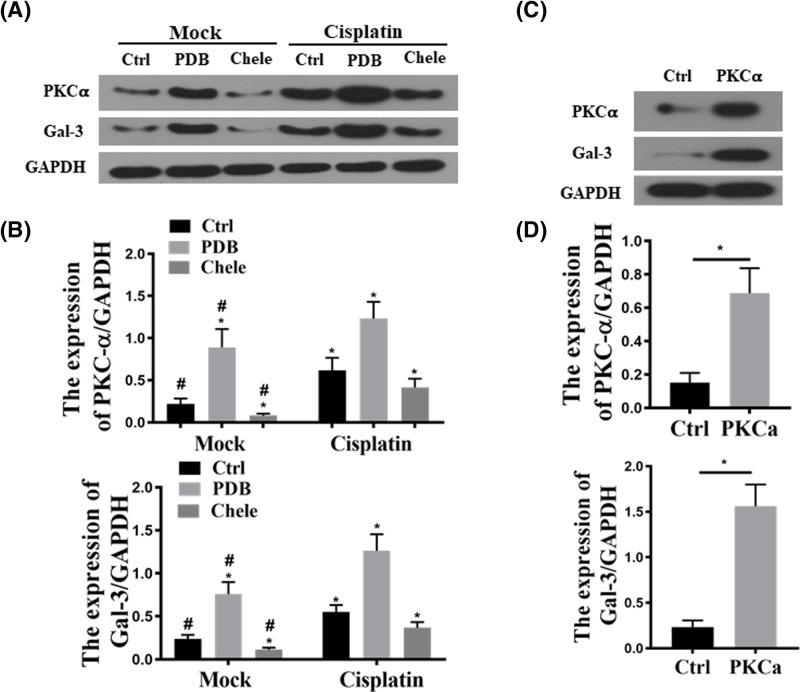
PKC-α mediated cisplatin-induced Gal-3 expression (**A**) HEK293 cells were pre-incubated with 2 μM PDB (Sigma–Aldrich, St. Louis, MO, U.S.A.) and 10 μM chelerythrine (che) for 30 min then treated with or without 2 μM cisplatin (CP) treatment. (**B**) The representative levels of PKC-α and Gal-3 in the HEK 293 cells. *P<0.05 vs Ctrl in Mock, ^#^*P*<0.05. (**C**) HEK293 cells were transfected with 2 μg/well of pcDNA3-PKC-α or vector alone. Gal-3 and PKC-α levels were examined by Western blot. (**D**) Quantification of Gal-3 and PKC-α levels based on Western blot; *P<0.05.

To explore the role of PKC-α and Gal-3 in the transition of AKI to CKD, we first treated HEK293 cells with PKC-α activator PDB and its inhibitor chelerythrine, at 30 min after treatment with cisplatin or saline. As shown in Figure 5A,B, PDB increased Gal-3 expression and chelerythrine reduced Gal-3 protein expression. Cisplatin treatment further enhanced the effects of PDB in inducing Gal-3 expression, while chelerythrine reduced cisplatin-stimulated Gal-3 up-regulation. To examine the interplay between PKC-α and Gal-3, PKC-α cDNA was transfected into HEK293 cells to monitor the change of Gal-3, which showed that overexpression of PKC-α increased Gal-3 levels (Figure 5C,D).

### Gal-3 blockage attenuated apoptosis and collagen I synthesis induced by PDB

**Figure 6:**
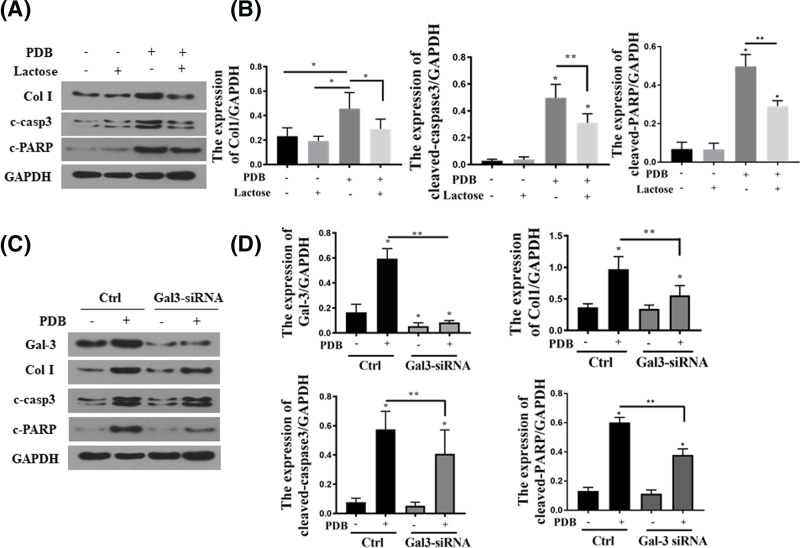
Block Gal-3 reduced PDB induced apoptosis and collagen I synthesis (**A** and **B**) The protein levels and representation of cleaved-caspase3, cleaved-PARP and collagen I in the HEK 293 cells (lactose 50 mM treated for 2 h); *P<0.05. (**C** and **D**) The protein levels and representation of gal-3, cleaved caspase-3, cleaved-PARP and collagen I in the HEK 293 cells; *P<0.05.

To determine whether PKC-α-stimulated collagen accumulation and apoptosis are mediated through Gal-3, cells were pre-treated with Gal-3 inhibitor, lactose, followed by PDB treatment. We chose lactose as the inhibitor as it competitively binds to the secondary structure of Gal-3, inhibiting Gal-3 activity [22]. As shown in Figure 6A,B, lactose blocked PDB-induced collagen I (Col I), cleaved-caspase-3 (c-casp-3) and c-PARP up-regulation. Similarly, Gal-3 knockdown by Gal3-siRNA transfection also resulted in the attenuation of Col I, c-casp-3 and c-PARP up-regulation induced by PDB (Figure 6C,D).

## Discussions

AKI and CKD are characterized by renal tubular cell apoptosis and tubular atrophy, leading to progressive loss of kidney functional decline. Cisplatin is a common chemotherapy drugs of cancer. One critical side effects of cisplatin is the renal toxicity, and one-third patients develop nephrotoxicity that necessitates immediate treatment [23]. AKI accounts for 1 to 25% of intensive care unit admissions and 1–7% of all hospital admissions. Moreover, AKI is a putative independent risk factor for mortality [24]. Progression of AKI to CKD is also common, which seriously compromises the life of quality of patients [25]. Here, we investigated the efficacy and mechanism of Gal-3 inhibition in attenuating cisplatin-induced kidney injury. Our results indicated that the Gal-3 inhibitor, MCP [26], markedly induced reduction of tubular damage induced by cisplatin, as evidenced by decreased Scr, improved tubular integrity and regeneration. Consistently, we showed that Gal-3 promotes apoptosis in HEK293. Indeed, previous evidences suggested that Gal-3 is a potential regulator of apoptosis [27,28]. However, in various tissues, Gal-3 may serve as both pro-apoptotic factor, e.g. through macrophage activation, macrophage phagocytosis [27], and anti-apoptotic factor, e.g. by blocking the intrinsic apoptotic intracellularly [28]. Our results suggest that Gal-3 inhibition attenuates kidney tissue apoptosis and confers protection against the progression of renal fibrosis.

We showed here that Gal-3 up-regulation, along with elevated expression of collagen I and fibronectin, was induced by cisplatin treatment. This observation is consistent with previous findings that interstitial matrix expansion coincides with tubular cell injury [29]. In renal fibrosis, the equilibrium of extracellular matrix synthesis and degradation is altered, resulting in elevated expression of matrix proteins. Excessive load of matrix protein is a crucial cause of tubulointerstitial inflammation, tubular cell injury and fibrosis. Gal-3 is a major player in ECM remodeling in kidney and was found to significantly contribute to progression of renal fibrosis via a variety of pathways [30]. In kidney transplantation, Gal-3 inhibition was also shown to reduce tubular atrophy and interstitial fibrosis [31]. Based on this, a number of strategies are directed toward inhibiting matrix synthesis through myofibroblasts targeting. We showed here that MCP-treated mice demonstrated reduced collagen I and fibronectin expression, which was in line with decreased ECM remodeling. Our results also echoed previous findings that Gal-3 plays an important role in intracellular matrix turnover via ECM interaction, supported by reduced collagen I and fibronectin content in kidney tissues after Gal-3 inhibition. Our results are in line with the pro-fibrotic and pro-inflammatory properties of Gal-3 in adipose tissue, which suggested that Gal-3 inhibition confers protective effects against adipose tissue remodeling [32]. Gal-3 inhibition has been applied for treated aldosterone-induced cardiac and renal injuries, owing to suppression of inflammation and matrix expansion [31]. Besides, Gal-3 inhibition was shown to effective sensitize cancers to cisplatin treatment [33]. This property could potential orchestrate with the renal protective effects to improve the clinical outcome of cancer patients.

We show that Gal-3 inhibition concurrently induced PKC-α suppression. PKC-α are a class of factors that contribute to renal tubular cell apoptosis [34]. The suppression of PKC-α is utilized in the management of chronic injury. Moreover, it has been demonstrated in mice that PKC-α deficiency could attenuate AKI and ischemia allograft injury [31]. However, to our knowledge, this is the first report on the involvement of PKC-α in cisplatin-induced AKI and CKD. The suppression of PKC-α can potentially shed light on the anti-apoptotic effects of Gal-3 inhibition in injured kidney. The interaction between Gal-3 and PKC-α has not been reported either. However, here we did not probe the molecular pathway associated with the interaction between Gal-3 and PKC-α, which should be a subject of further research. Moreover, the efficacy of Gal-3 inhibition in other AKI models, such as glycerol-induced model, ischemia–reperfusion model etc. is also worth testing to fully characterize this therapy in renal protection.

## Conclusions

In summary, the present study on Gal-3 inhibition is a viable strategy for alleviating AKI and CKD transition induced by cisplatin. This effect is mediated by the reduction of cell apoptosis and attenuation of collagen I and fibronectin. Gal-3 inhibition also suppresses PKC-α. Therefore, Gal-3 and PKC-α are potential therapeutic targets for the treatment of AKI and CKD. Further development of Gal-3 and PKC-α inhibition strategies could overcome nephrotoxicity of drugs and improve the therapeutic outcome of other kidney diseases.

## Supporting information

**Supplementary Figure S1:** Inflammatory factors levels in vivo and in vitro. A. The levels of proinflammatory mediators including interleukin (IL)-1b, interleukin-6, tumor necrosis factor (TNF)-a, and monocyte chemoattractant protein (MCP)-1 measured by enzyme-linked immunoabsorbent assay in different group mice. B. Relative mRNA levels of proinflammatory mediators in HEK293 cells.

## Abbreviations

AKI: acute kidney injury
CKD: chronic kidney diseases
ECM: extracellular matrix
Gal-3: galectin-3
MCP: modified citrus pectin
PKC-α: protein kinase C α
