# Supplementary material for: Galectin 3 inhibition attenuates renal injury progression in cisplatin-induced nephrotoxicity
Source: Biosci Rep. 2018 Dec 18;38(6):BSR20181803. doi: 10.1042/BSR20181803 (PMC6435560; doi:10.1042/BSR20181803)
Supplement: Supplementary file 1 [file bsr20181803_Supp1.pdf]

S1. Inflammatory factors levels in vivo and in vitro. A. The levels of proinflammatory mediators including interleukin (IL)-1b, interleukin-6, tumor necrosis factor (TNF)-a, and monocyte chemoattractant protein (MCP)-1 measured by enzyme-linked immunoabsorbent assay in different group mice. B. Relative mRNA levels of proinflammatory mediators in HEK293 cells.

S1.

A.

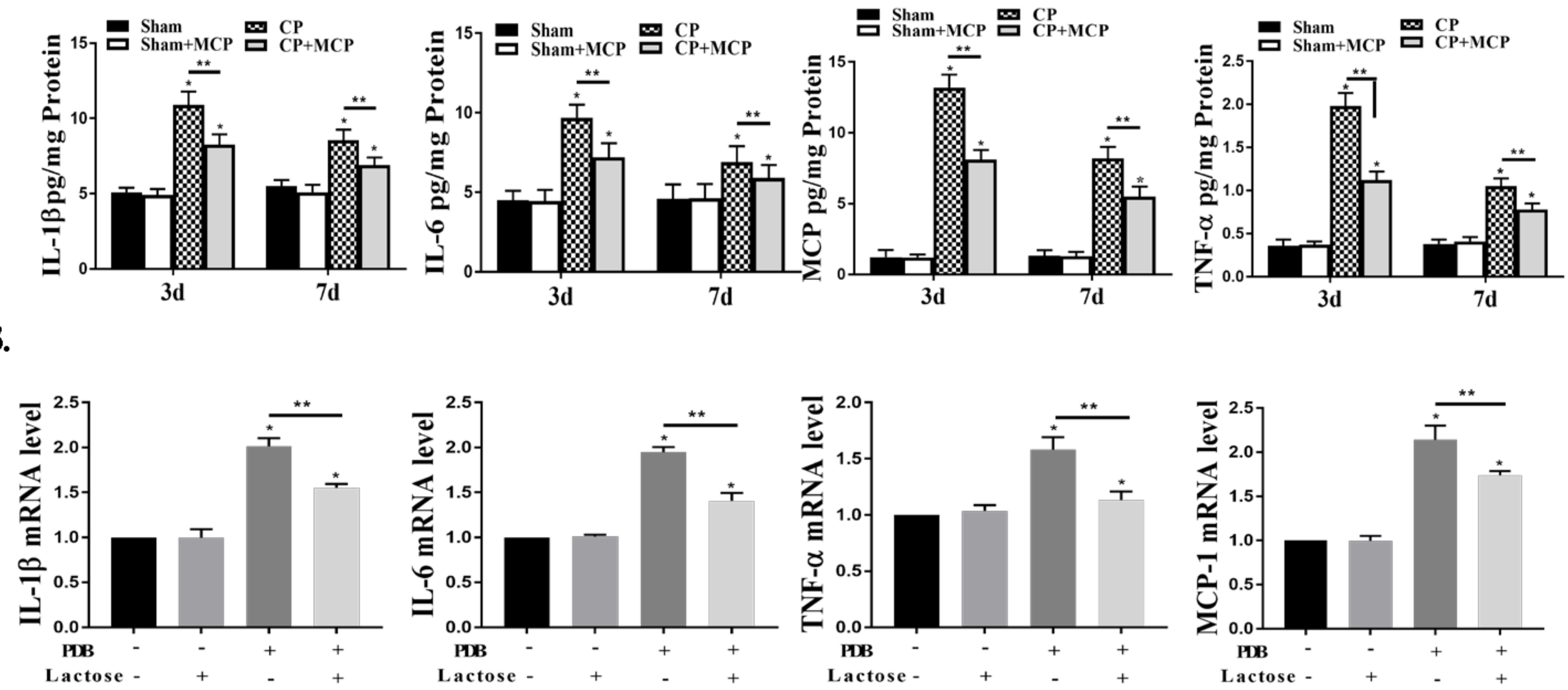

Sham
Sham+MCP
CP
CP+MCP
IL-1β pg/mg Protein
IL-6 pg/mg Protein
MCP pg/mg Protein
TNF-α pg/mg Protein
3d
7d
B.
IL-1β mRNA level
IL-6 mRNA level
TNF-α mRNA level
MCP-1 mRNA level
PDB - - + +
Lactose - + - +
